# A Mark-Release-Recapture Study to Estimate Field Performance of Imported Radio-Sterilized Male *Aedes albopictus* in Albania

**DOI:** 10.3389/fbioe.2022.833698

**Published:** 2022-08-16

**Authors:** Enkelejda Velo, Fabrizio Balestrino, Përparim Kadriaj, Danilo Oliveira Carvalho, Ahmadou Dicko, Romeo Bellini, Arianna Puggioli, Dusan Petrić, Antonios Michaelakis, Francis Schaffner, David Almenar, Igor Pajovic, Alfred Beqirllari, Migel Ali, Gjergji Sino, Elton Rogozi, Vjola Jani, Adelina Nikolla, Tanja Porja, Thanas Goga, Elena Fălcuă, Mihaela Kavran, Dubravka Pudar, Ognyan Mikov, Nadya Ivanova-Aleksandrova, Aleksandar Cvetkovikj, Muhammet Mustafa Akıner, Rados Mikovic, Lindita Tafaj, Silva Bino, Jeremy Bouyer, Wadaka Mamai

**Affiliations:** ^1^ Department of Epidemiology and Control of Infectious Diseases, Institute of Public Health, Tirana, Albania; ^2^ Joint FAO/IAEA Programme of Nuclear Techniques in Food and Agriculture, Vienna, Austria; ^3^ Centro Agricoltura Ambiente (Italy), Crevalcore, Italy; ^4^ Statistics for Development–STATS4D, Sacre Coeur III, Dakar, Senegal; ^5^ Faculty of Agriculture, University of Novi Sad, Vojvodina, Serbia; ^6^ Scient.Directorate of Entomology and Agricultural Zoology, Benaki Phytopathological Institute, Kifissia, Greece; ^7^ Francis Schaffner Consultancy, Riehen, Switzerland; ^8^ Empresa de Transformación Agraria S.A., S.M.E, M.P. (TRAGSA), Paterna, Spain; ^9^ Biotechnical Faculty, University of Montenegro, Podgorica, Montenegro; ^10^ Invent” Ltd., Tirana, Albania; ^11^ Local Health Care Unit of Lezhë, LezhÃ, Albania; ^12^ Department of Physics, Faculty of Natural Sciences, “MeteoAlb” Politechnic University of Tirana, Tirana, Albania; ^13^ Aide to the Prime Minister, Albania Department of Risk Communication and Community Engagement, WHE Balkan Hub, WHO Regional Office for Europe, Belgrade, Serbia; ^14^ “Cantacuzino” National Military-Medical Institute for Research and Development, Bucharest, Romania; ^15^ National Centre of Infectious and Parasitic Diseases, Sofia, Bulgaria; ^16^ Department of Parasitology and Parasitic Diseases, Faculty of Veterinary Medicine-Skopje, Ss. Cyril and Methodius University in Skopje, Skopje, North Macedonia; ^17^ Department of Biology, Faculty of Arts and Sciences Department of Biology, Recep Tayyip Erdogan University, Rize, Turkey; ^18^ Veterinary Diagnostics Laboratory, Podgorica, Montenegro; ^19^ Institute for Agricultural Research for Development (IRAD), Yaounde, Cameroon

**Keywords:** mosquitoes, pest, management, survival, dispersal, competitiveness, BG sentinel trap, Sterile Insect Technique

## Abstract

The pathogen transmitting *Aedes albopictus* mosquito is spreading rapidly in Europe, putting millions of humans and animals at risk. This species is well-established in Albania since its first detection in 1979. The sterile insect technique (SIT) is increasingly gaining momentum worldwide as a component of area-wide-integrated pest management. However, estimating how the sterile males will perform in the field and the size of target populations is crucial for better decision-making, designing and elaborating appropriate SIT pilot trials, and subsequent large-scale release strategies. A mark-release-recapture (MRR) experiment was carried out in Albania within a highly urbanized area in the city of Tirana. The radio-sterilized adults of *Ae. albopictus* Albania strain males were transported by plane from Centro Agricoltura Ambiente (CAA) mass-production facility (Bologna, Italy), where they were reared. In Albania, sterile males were sugar-fed, marked with fluorescent powder, and released. The aim of this study was to estimate, under field conditions, their dispersal capacity, probability of daily survival and competitiveness, and the size of the target population. In addition, two adult mosquito collection methods were also evaluated: BG-Sentinel traps baited with BG-Lure and CO_2,_ (BGS) versus human landing catch (HLC). The overall recapture rates did not differ significantly between the two methods (2.36% and 1.57% of the total male released were recaptured respectively by BGS and HLC), suggesting a similar trapping efficiency under these conditions*.* Sterile males traveled a mean distance of 93.85 ± 42.58 m and dispersed up to 258 m. Moreover, they were observed living in the field up to 15 days after release with an average life expectancy of 4.26 ± 0.80 days. Whether mosquitoes were marked with green, blue, yellow, or pink, released at 3.00 p.m. or 6.00 p.m., there was no significant difference in the recapture, dispersal, and survival rates in the field. The Fried competitiveness index was estimated at 0.28. This mark-release-recapture study provided important data for better decision-making and planning before moving to pilot SIT trials in Albania. Moreover, it also showed that both BG-traps and HLC were successful in monitoring adult mosquitoes and provided similar estimations of the main entomological parameters needed.

## Introduction

Mosquitoes represent a threat to both human and animal health. They are vectors of various diseases such as malaria, dengue, chikungunya, Japanese encephalitis, West Nile virus, Rift Valley fever, yellow fever, and Zika and lymphatic filariasis (Tolle, 2009; Kampen et al., 2012).

The invasive tiger mosquito *Aedes (Stegomya) albopictus* (Skuse, 1895), native to Southeast Asia, has colonized all continents (Benedict et al., 2007). It was introduced to Europe at the end of the 20th century (Lounibos, 2002; Scholte and Schaffner, 2007; Medlock et al., 2012), and its first occurrence was reported in Albania in 1979 (Adhami and Reiter, 1998). The species is now well-established and present even in tiny isolated villages in high-altitude (>1,200 m) environments (Tisseuil et al., 2018). Although the risk for pathogen transmission related to the autochthonous *Aedes* species is currently considered low, intensified vector control measures are needed to prevent disease outbreaks similar to those that occurred in various countries with unexpected local cases of chikungunya, dengue, and Zika (ECDC, 2019; Vermeulen et al., 2020). Even in the absence of disease transmission, *Ae. albopictus* is a significant nuisance species in urban areas (Kolimenakis et al., 2019).

Since its first report from Albania, it has been recorded in numerous European countries (Medlock et al., 2012; Medlock et al., 2015) and became well-established across Mediterranean countries, and more recently it spread northward to Germany (Becker et al., 2017), North Macedonia (Cvetkovikj et al., 2020) and Moldova (Șuleșco et al., 2021), and Austria (Schoener et al., 2019; Bakran-Lebl et al., 2021).

European health officials are concerned about the risk posed by invasive mosquito species to public health, and the World Health Organization (WHO) office for Europe is suggesting to rapidly develop the required capacities to face the problem (Bellini et al., 2020). Due to the rapid spread of resistance to commonly used insecticides (Hemingway and Ranson, 2000; Vontas et al., 2012; Grigoraki et al., 2017; Pichler et al., 2018), integrated vector management including new methods is now widely accepted, and this strategy is emphasized in the Global Vector Control Response (WHO, 2017). The sterile insect technique (SIT), an insect birth control method, has historically been used to suppress and even eradicate several agricultural and livestock/human pests (Vreysen et al., 2000; Dyck et al., 2021). In response to increasing demand for SIT application from the International Atomic Energy Agency (IAEA)/Food and Agriculture Organization of the United Nations (FAO) Member States, substantial efforts have been invested in the development of the SIT package against mosquitoes including the development of equipment and protocols for mass-rearing, sex-separation, irradiation, handling, packing, transport, release, and quality control (Balestrino et al., 2014a; Balestrino et al., 2014b; Bimbilé-Somda et al., 2019; Culbert et al., 2019, 2020; FAO/IAEA, 2020b; Maiga et al., 2016; Maïga et al., 2017; Maïga et al., 2019; Mamai et al., 2017; Mamai et al., 2019a; Mamai et al., 2019b; Yamada et al., 2019; Zheng et al., 2015). The WHO and IAEA have recently published a joint guidance framework for testing SIT as a vector control tool against *Aedes*-borne diseases (WHO/IAEA, 2020).

Understanding the bio-ecological features of the target population and how laboratory-produced sterile males may perform in the natural environment is crucial. Mark-release-recapture (MRR) studies are particularly useful and have been frequently applied to various insect species to study characteristics of populations related to the ecology, biology, behavior, ability to transmit pathogens, and ultimately their control (Gillies, 1961; Pollock, 1991; Hagler and Jackson, 2001; Silver, 2007; Bellini et al., 2010; Epopa et al., 2017; Benedict et al., 2018; Oliva et al., 2021). Knowledge of the characteristics of sterile males and reliable quantification of wild population density are prerequisites for planning SIT interventions (Bouyer et al., 2020b; Romeis et al., 2020; Oliva et al., 2021). The few MRR studies available for radio-sterilized *Ae. albopictus* males (Iyaloo et al., 2020) and non-radio-sterilized males (Le-Goff et al., 2019) mainly aimed at assessing survival, dispersal, and/or population size analysis. Only four field estimations of competitiveness are currently available (Zheng et al., 2019; Bouyer et al., 2020a; Iyaloo et al., 2020; Bellini et al., 2021). An appropriate method for population monitoring is also necessary to apply SIT. Although the BG-Sentinel trap (BGS) is considered the gold standard method for catching *Aedes* mosquitoes (Williams et al., 2006; Farajollahi et al., 2009; Staunton et al., 2020), some factors including shade, presence of bushes, and potential larval habitats were shown to influence its efficacy (Staunton et al., 2020).

In this study, we investigated the performance of a radio-sterilized local strain of *Ae. albopictus* using MRR. Specifically, parameters that were assessed included 1) recapture rate, 2) probability of daily survival, 3) dispersal capacity, 4) sterile-to-wild male ratio, 5) wild population estimation, and 6) field competitiveness. We also exploited the data to compare the efficiency of two adult mosquito trapping methods to estimate these parameters, namely, the BG-Sentinel 2™ used with BG-Lure^®^ and CO_2_ and the human landing catch.

## Material and Methods

### Study Site

**FIGURE1:**
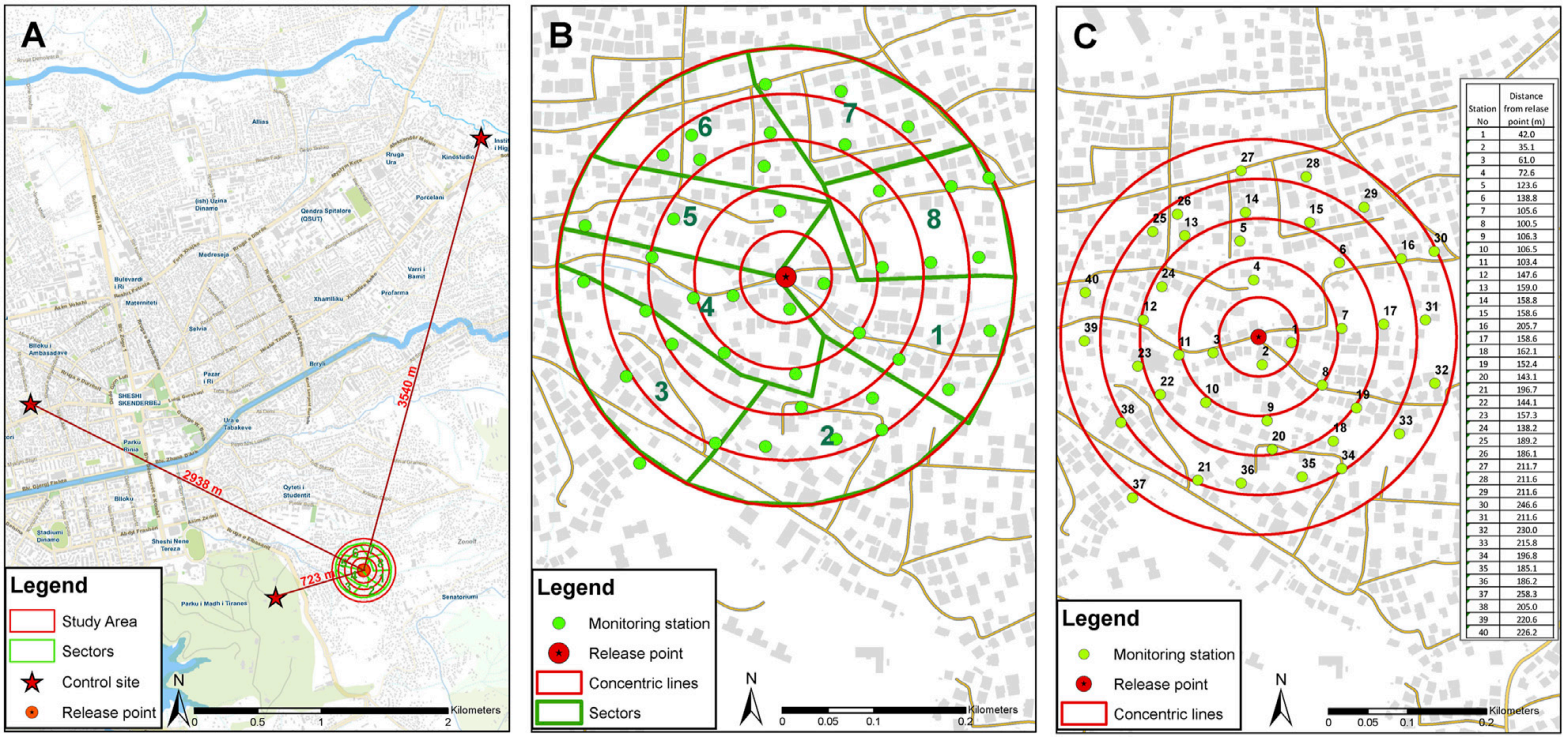
Map of mark release recapture setup in Tirana and distribution of 40 trapping stations for mosquito collection (two stations per hectare). **(A)** Location of release and control sites. **(B)** Study area is divided into sectors for simultaneous monitoring by different teams (green lines). The red stars represent the control sites, 700, 3,040, and 3,440 m from the release site. **(C)** Positions and number of trapping stations. Concentric red lines represent five annuli at 50, 100, 150, 200, and 250 m from the release point (red point in the center).

The MRR study was conducted in an urban area of Tirana (41°19′44″N, 19°49′04″E), the capital and the largest city of Albania. The presence and establishment of *Ae. albopictus* in the area have been proven via monitoring activities since 2010. The area is characterized by two-storied houses with many private and some public gardens (Figure 1). The field monitoring was conducted by ovitraps following the standard operating procedure by Bellini et al. (2021), with slight modifications (filter paper instead of Masonite paddle as oviposition substrate). The egg monitoring started in May 2017 (week 22) before the sterile male releases, to allow the required field data collection on population dynamics and egg fertility (unpublished data). Fertility of wild eggs was assessed by standardized hatching procedures (see Supplementary material S1) during the MRR trials on egg samples collected both in the release area and in separated control areas (700 m West-Southwest, 3040 m Northwest, and 3440 m North from the release site) with comparable land use and cover.

### Origin of the Mosquitoes and Rearing Procedures

Mosquitoes released in this MRR experiment were obtained from *Ae. albopictus* eggs collected in Albania (ALB strain) and amplified in the biosafety level 2 (BSL-2) laboratory of the Medical and Veterinary Department of the Environmental and Agriculture Centre “G. Nicoli” at CAA (Bologna, Italy). Research carried out on mosquitoes in confined laboratory conditions do not require a specific permit according to the directive 2010/63/EU of the European Parliament and of the Council on the protection of animals used for scientific purposes. Adult mosquitoes were maintained following environmental and rearing conditions described in Balestrino et al. (2014b). The second generation (F2) was sent to Albania for the MRR study.

### Sexing and Transportation

Sex separation was carried out according to the standard method of sieving, at 24–30 h from the beginning of pupation (Bellini et al., 2007; Medici et al., 2011). The residual amount of females in male pupae was 1%. The pupae collected were aliquoted in batches of 2,000 pupae each and transferred into plastic containers of 500 ml capacity filled with 200 ml of water. These containers were used to transport the pupae to the Medical Physics Department of the St. Anna Hospital (Cona, Ferrara, Italy) for irradiation and brought back afterward to the laboratory for packaging and adult emergence. During transportation, the pupae were maintained in thermal insulated plastic containers with changing phase materials (PCM ice gel packs; Blu Ice, Dryce Srl, Milano, Italy) to maintain a temperature of about 15°C.

### Irradiation Treatments

Irradiation treatments were performed using an IBL 437 irradiator (CIS Bio International, Bagnols-sur-Ceze, France) equipped with a 50.9 TBq Cs-137 linear source with a central dose rate of 1.8 Gy/min. At the hospital, the different batches of pupae were transferred into separated petri dishes (12 cm diameter) filled with 25–30 ml of water and piled inside a dedicated canister for irradiation (Balestrino et al., 2010). The dose of gamma rays administered to the pupae was equal to 40 Gy.

### Packaging and Shipment

At the CAA laboratory, the irradiated pupae inside petri dishes with water (25–30 ml) were placed at the bottom of cardboard boxes (12cm^3^ × 12cm^3^ × 18 cm^3^) closed at the top with a mosquito net for emergence and transportation. Cotton pads soaked with 10% sugar solution were provided and secured at the top of each box to assure adult nourishment. Additional separator partitions were added inside each box to provide an overall vertical resting surface area of 1.04 cm^2^ per adult. The mosquito boxes were maintained at about 20°C for the first two days after adult emergence, and after removing water from the petri dishes, the boxes were transferred inside a larger polystyrene container with an adequate quantity of ice gel packs to maintain a temperature between 10 and 15°C during transportation to the release site. In order to maintain this temperature range over 24–48 h, we used a 70-L polystyrene container filled with 10 kg of ice gel packs conditioned at 4°C (PCM Blu Ice) and 2 kg at −20°C (PCM Green Ice, Dryce Srl, Milano, Italy). A data logger was also introduced inside each container to register the environmental conditions during transport.

### Mosquito Marking Procedure

Within 3 h of reception, the males were sugar-fed and marked with fluorescent dust (RADGLO^®^ JST, Radiant NV, Houthalen, Belgium) (FAO/IAEA, 2020a) applied using a bulb duster to create a dust storm within the transportation cardboard boxes (Supplementary material S2). A fixed dose of fluorescent powder equal to 0.6 gr (0.3 gr per 1,000 adult) was used per each cardboard box and manually insufflated to disperse the powder uniformly on mosquitoes just before the release. Culbert et al. (2020) have shown that with the current dose of the fluorescent dust used, there was no effect on the survival of *Aedes* mosquitoes. Fluorescent dust coverage on male body parts was evaluated on a sample of about 300 mosquitoes randomly collected from the different cardboard boxes upon each release.

### Mosquito Release and Recapture

The MRR study was undertaken from 4th September to 2nd October 2017. One release of sterile marked males per week was performed using different colors on two consecutive weeks. The mosquito release point was located in the center of the 20-ha study area (Figure 1), where 40 sites were selected to sample the mosquito population homogenously (two sampling stations per hectare). The sampling sites were arranged the day before the first males’ release in five successive concentric circles placed 50 m apart starting from the central release point (Figure 1A). In each sampling station, a pair of traps were set and spaced 10–20 m apart, consisting of a BG-Sentinel 2™ (Biogents, Regensburg, Germany—BGS) baited with dry ice (1 kg/24 h) and BG-Lure™ (Biogents, Regensburg, Germany) and an ovitrap made from a 500-ml black polypropylene cup (Luwasa Interhydro AG, Allmendingen, Switzerland; 11/9 hydroculture pot; 11 cm diameter x 9 cm high) lined with heavy-weight seed germination paper (#76 seed germination paper, Extra Heavy Weight, Anchor Paper Co., St Paul, MN, United States). Ovitrap and BGS placed in the same sampling station were coded with the same station number.

Right after marking (Supplementary material S2), the mosquito males were transferred to the center of the study area for release. The dusted males were released as young adults (72–96 h-old) by placing them on the ground and opening the cardboard boxes in a shaded area. The boxes were gently shaken to induce the males to exit. The males that remained in the cage after 30 min were considered dead. The two releases of *Ae. albopictus* sterile males were in the amount of ca 30,000–32,000 males each. The release dates were September 6 (16,000 orange marked sterile males at 3:00 p.m. plus 16,000 green marked sterile males at 6:00 p.m.) and September 13 (16,000 yellow marked sterile males at 3:00 p.m. plus 14,000 pink marked sterile males at 6:00 p.m.). Eight teams were involved in this study. Teams included expert entomologists who decided the exact position of each trap in the field according to the available environment characteristics, prefered by *Ae. albopictus*. Each team was in charge to place and daily inspect four to six stations. Starting from the first trap positioning and during every visit, each team collected *Ae. albopictus* adult males and females flying around the team members (human landing catch, HLC) by handmade manual battery aspirators (12 V DC, 0.19 A, aspiration 2.5 m/s) (Balestrino et al., 2017) for 15 min in each sampling station. Only one operator per team performed the HLC, and the collected adults were recorded with an identification code referring to the sampling station and the collection date. The collection of samples (HLC, BGS, and ovitrap) from different sites was conducted every day from 3:00 p.m. to 6:00 p.m., except on Sunday. In case of unfavorable weather conditions (rainfalls), the HLC was not conducted. The sequence of stations was randomly rotated daily at each visit to avoid possible collection time bias. Each team was provided with an insulated thermal container to transport dry ice and store and euthanize the adult samples collected (during the daily collection). Collected adult mosquitoes were stored overnight and screened for identification of species and coloration the following day under a stereomicroscope and using a 12-V UV lamp. The use of a UV light source was to facilitate the identification of fluorescent dust on the male mosquito collected.

Egg papers collected from ovitraps were stored in plastic bags (not completely sealed) and transported at ambient temperature. The number of eggs on each paper was counted the following day, and the portion of the paper with eggs was cut out in strips, stored, maturated, and hatched at least 7 days after the collection day according to the procedures in Supplementary Material S1A. Each day after collection, the adult field data samples were analyzed, classified, and stored into a Geographical Information System platform to calculate the distances between release and each recapture site. In this trial, a dedicated WebGIS application available at https://invent.al/produkte/index.html under the MRR training link was used to georeference and manage the field data collected. BGS were stopped after two trapping sessions without any collection of marked males, and after this date, only ovitraps were monitored for 4 additional weeks. The activity continued this scheme until October 02, which was 19 days after the last release. Weather parameters were collected from a meteorological station located in the neighborhood (for rainfall and wind speed) (see Supplementary Material Figure S1; Supplementary Table S1). In parallel, in three other areas nearby, where no sterile males were released (control areas, see Figure 1A), five ovitraps were positioned in each and checked weekly to estimate the natural egg fertility and to measure possible failure to hatch caused by diapause.

### Statistical Analysis

Statistical analyses were performed using R Software version 4.1.0 (R Development Core Team 2008; https://www.R-project.org/). A generalized linear model (with Poisson family distribution and log as link function) was used to check for difference in the main parameters between trapping methods, marking colors, and time of release. For the difference in the natural egg fertility between the control sites, we used a binomial generalized linear mixed model fit by maximum likelihood (Laplace Approximation) with egg hatch as the response variable and the ovitrap station as a random effect. The male dispersal has been analyzed by the mean distance traveled (MDT), the maximum distance traveled (MAX), and the flight range (FR). Dispersal distance estimation of *Ae. albopictus* males was facilitated by the homogeneous density of recapture stations in the study area. The FR was estimated through the linear regression of the cumulative estimated recaptures performed in each recapture stations (*x*-axis) on the log10 (annulus median distance). The FR_50_ and FR_90_ indicate the distance that comprehends the maximum flight distance reached by 50% and 90% of the individuals, respectively. These parameters were calculated from the equation of regression as the value of the *y* axis at 50% and 90% of the largest value of x, respectively. A random isotropic model in two dimensions has also been fitted to the data to calculate the diffusion coefficient.

The survival rate of sterile males was estimated by the linear corrected method (Harrington et al., 2001; Buonaccorsi et al., 2003) as follows: *θ* = *e* ^α^/(N+ *e* ^α^), S = *e* ^b^/(1- *θ*)^1/d^, where a and b are the regression coefficients of the linear regression of the log-transformed captures as a function of time, N is the number of individuals released, *θ* is the recapture rate, d is the number of days after release, and s the survival rate. The probability of daily survival (PDS) in the field is estimated by regressing log_10_ (x +1) of the number of recaptures against the day of recapture where the antilog_10_ of the slope of the regression line is the PDS (Muir and Kay, 1998). Average life expectancy in the field (ALE) is calculated from the PDS as 1/- log_e_ PDS.

Based on the sterile males released and recaptured, the wild male population size in the study area was estimated using the modified Lincoln index that corrects for small samples and compensates for daily survival P = [R*S (n − m + 1)]/(m + 1), where R is the number of originally marked males, S is the daily survival rate, n is the total number of recaptures of both marked and wild adult males, and m is the number of recaptured marked males. These data, together with the fertility rate of the eggs (hatched eggs and normally shaped eggs with the presence of embryo are considered fertile) collected in the release and control sites allow us to estimate the sterile male competitiveness index under field conditions using the Fried competitiveness index (Fried, 1971) as follows: F = ((Ha–Ee)/Ee)/R, where Ha = natural fertility in the control site (determined during the MRR feasibility study period) and Ee = observed fertility rate, R = ratio of sterile over wild males. Using data from BGS, a nonparametric bootstrap approach (Efron, 1979) was applied to obtain a confidence interval for the estimate of the Fried index as described in Bouyer et al. (2020a). In brief, the data on fertility and the ratio of sterile males over wild ones were resampled without replacement, and for each set of resampled data, the Fried index was computed (1,000 simulations). Assuming a symmetric distribution, the basic percentile method to get a 95% confidence interval was used.

## Results

### Environmental Weather Conditions in the Study Area During the Mark-Release-Recapture Experiment

The mean daily temperature in Tirana for the whole 2017 years ranged from −3.46°C to 33.51°C (mean ± se = 17.65 ± 0.40°C). The environmental weather data variation and the average of the main variables over the MRR experimental period (September 2017 and October 2017) are presented in Supplementary Material Table S1 ; Supplementary Figure S1). The mean (daily) temperature during the MRR ranged from 12.46 to 27.08 C (mean ± se = 19.60 ± 0.44°C). The mean daily relative humidity for 2017 ranged from 32.69% to 100% (mean ± se = 60.74% ± 0.66%). For September and October, the mean relative humidity ranged from 47.07% to 84.14% (mean ± se = 64.43 ± 1.03%). No rainfall was recorded during the two days before the first release. However, the day before the second release, there were strong rainfalls followed by minor rainfalls during the day of release. The daily wind speed was constant (Supplementary Table S1), ranging from 0.9 to 1.5 m/s.

### Recapture of *Aedes albopictus* and Trap Efficiency

Out of the estimated 62,000 color-marked sterile males, a total of 48,011 flew from the release boxes and 1,887 were recaptured throughout the 3-week collection period by both methods, representing 3.93% of the total males released (Table 1). Out of these recaptured sterile males, 59.94% were obtained from BGS (2.36% of the total males released), while the remainder 40.06% (1.57% of the total males released) from HLC. Considering the entire period of collection, there were no statistical difference in the recapture rates of sterile males between the trapping methods BGS and HLC (GLM, df = 1, F = 3.53, *p* = 0.06) and dust marking colors (GLM, df = 3, F = 0.406, *p* = 0.7490). However, total captures of wild males and females were much higher with BGS than with HLC (GLM, df = 1, F = 11.23, *p* = 0.0008 and df = 1, F = 54.12, *p* = 2.13e-13 for males and females, respectively). Moreover, the time of release (3:00 p.m. or 6:00 p.m.) did not impact the recapture rate of the released sterile males (GLM, df = 1, F = 0.836, *p* = 0.361).

**TABLE 1:** Number of released and recaptured radio-sterilized males *Aedes albopictus*.

Color	Release date	Time of release	Initial males delivered and marked	Mortality before release (%)	Males that flew	Number (%) recaptured with BGS	Number (%) recaptured with HLC	Number (%) total recaptured
Green	06/09/2017	3:00 p.m.	16,000	24.04	12,124	222 (1.83)	102 (0.84)	324 (2.67)
Orange	06/09/2017	6:00 p.m.	16,000	27.58	11,558	288 (2.49)	158 (1.37)	446 (3.86)
Yellow	13/09/2017	3:00 p.m.	16,000	22.66	12,344	325 (2.63)	269 (2.18)	594 (4.81)
Pink	13/09/2017	6:00 p.m.	14,000	14.39	11,985	296 (2.47)	227 (1.89)	523 (4.36)
Total			62,000	22.56	48,011	1,131 (2.36)	756 (1.57)	1887 (3.93)

**FIGURE 2:**
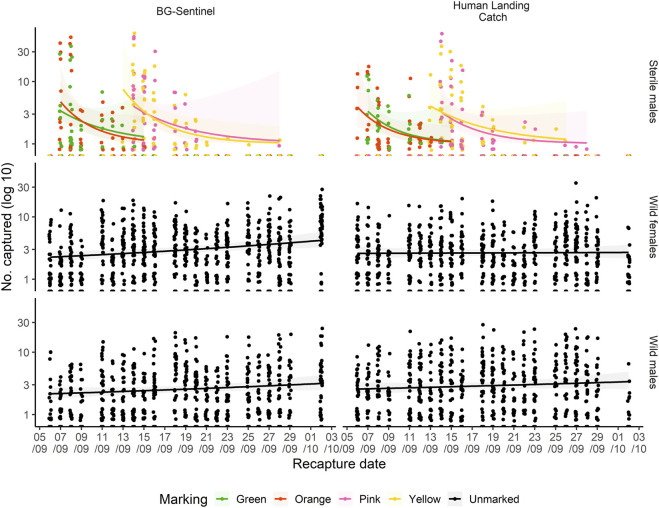
Capture of dusted-marked sterile males, wild male and female (both unmarked) mosquitoes as a function of time (September and October 2017) elapsed since release using BG-Sentinel trap and human landing catch. The regression line shows the trend of increase/decrease.

Figure 2 shows variation in the recapture of sterile males as well as of wild mosquitoes with both methods as a function of time following the releases. Overall, significant variation was observed in mosquito recaptures over days of collection and the distances from the release point. The number of recaptured mosquitoes declined over time. The majority (76.47%, *n* = 1,443) of the total recaptured (*n* = 1887) mosquitoes was collected shortly after their release (the first two days post-release), 93.64% (*n* = 1767) within 5 days post-release and no mosquito was recaptured beyond 15 days post-release (Figure 2).

As sex separation before release is not always 100% efficient, there was a chance that up to 1% of females could have been released with males. In this study, 11 irradiated females that were released with males were recaptured (three with HLC and eight with BGS). Among them, one female was recaptured 247 m from the release site 1 day after release and one recaptured at 212 m 7 days after release.

### Dispersal and Survival

**FIGURE 3:**
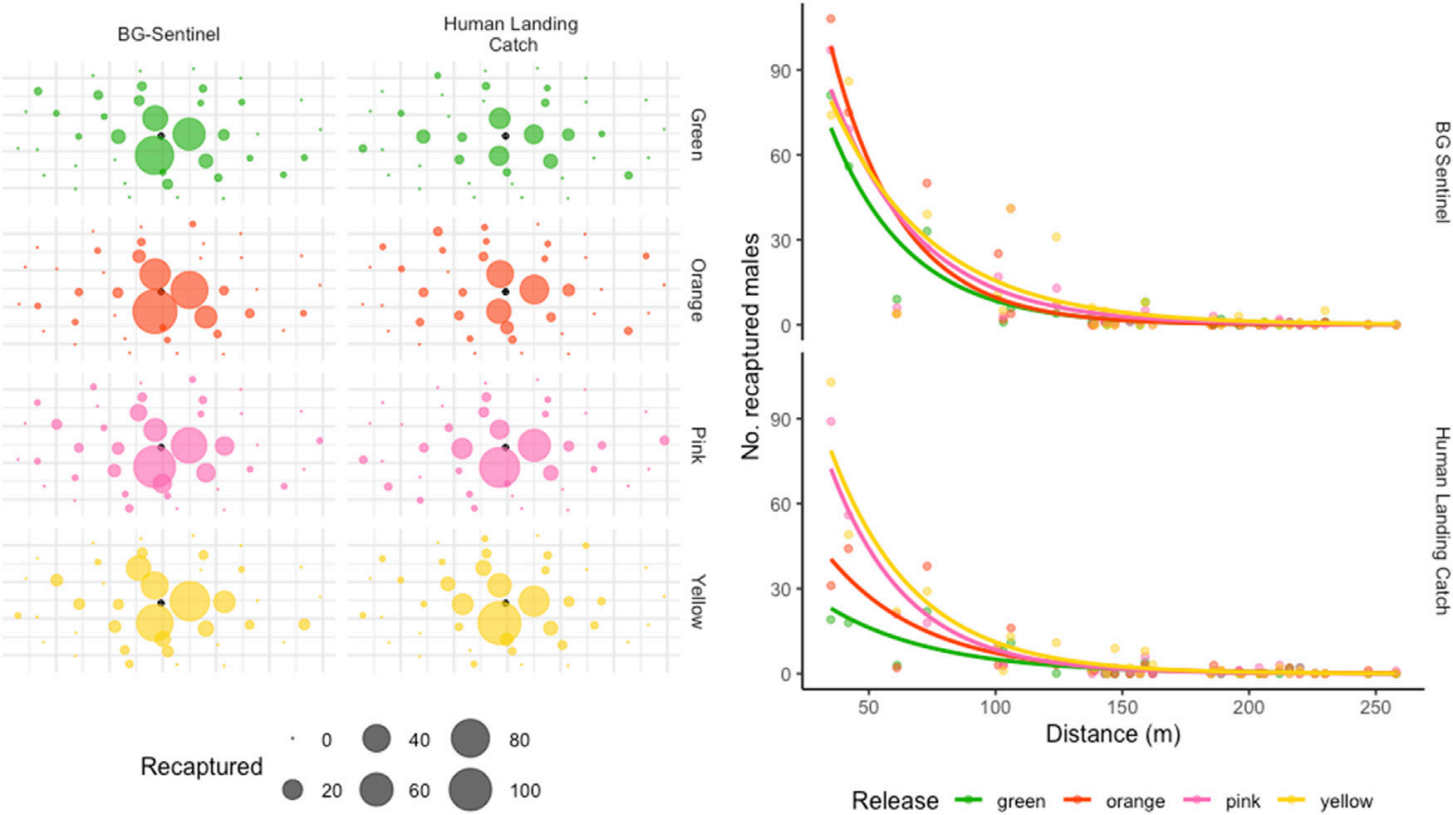
Distribution of accumulated recaptured sterile males in different collection stations (Figure 3A) and dispersal pattern as a function of distance from the release point (Figure 3B). The black dot in Figure 4A represents the release point, while the size of the dots corresponds to the number of marked mosquitoes caught at each site.

Figure 3 shows the recapture of sterile males as a function of distance from the release point. The dispersion pattern around the released point of the recaptured population remained similar between colors and trapping methods (Figure 3A). The number of recaptured marked males declined with distance from the release point (Figure 3B). About 55.93% of sterile males were caught within a 50 m radius, 73.24% within 100 m, and 90% within 125 m from the release site (Figure 1). Only 1.75% was caught between 200 and 250 m radius (there were no traps beyond the 258 m). One sterile male was captured (HLC) at 258 m from the single release point 6 days after release, and one sterile male (HLC) was recaptured the day of its release at 247 m from the release point.

The dispersal pattern, estimated by the MDT, MAX, and FR within which 50% or 90% of mosquitoes are expected to disperse (FR_50_ and FR_90_, respectively), is presented in Table 2. Overall, the released males were estimated to travel a mean ± SD of 93.85 ± 42.58 m (the estimated MDT over the entire period was 104.15 ± 41.95 m, 87.6 ± 32.6 m, 95.55 ± 55.5 m and 89.1 ± 40.4 (mean ± SD) m for green, orange, pink and yellow color marked males respectively). However, based on collections, the MDT was 91.2 ± 40.25 m and 96.8 ± 48.8 m for BGS and HLC, respectively. The maximum flight distance reached by 50% and 90% of the individuals was 33.01 m and 141.95 m, respectively.

**TABLE 2:** Mean distance traveled (MDT), the maximum distance traveled (MAX), and flight range of 90% (FR_90_) and 50% (FR_50_) of the radio-sterilized *Aedes albopictus* males in the field.

Marked sterile males	Trapping method	MDT (m)	MAX (m)	FR_50_ (m)	FR_90_ (m)
Green	BGS	99.3 (48.8)	230	27.2	131.1
	HLC	109 (35.1)	220	47.7	152.2
Orange	BGS	76.7 (23.4)	230	25.7	125.3
	HLC	98.5 (41.8)	247	40.0	141.4
Pink	BGS	85.1 (41.4)	216	28.1	138.9
	HLC	104 (69.6)	258	28.8	163.3
Yellow	BGS	98.3 (41)	230	35.6	148.2
	HLC	79.9 (39.5)	220	31.0	135.2

BGS, BG-Sentinel trap; HLC, Human Landing Catch. Values in parentheses represent the standard deviation.

The results of daily survival probability (PDS) and average life expectancy (ALE) of color-marked sterile males are presented in Table 3. Regardless of the color of the trapping method used, the ALE in the field varied from 3.03 to 5.76 days with average of 4.17 and 4.35 days for BGS and HLC, respectively. No statistical significance difference was found between BGS and HLC (GLM, df = 1, F = 0.213, *p* = 0.676). The PDS were also similar between BGS and HLC, varying from 0.72 to 0.84 with a mean of 0.79 for both methods and no statistical significance (GLM, df = 1, F = 0.02, *p* = 0.984). The four colors utilized showed no statistical difference both in PDS (GLM, df = 3, F = 4.437, *p* = 0.126) and ALE (GLM, df = 3, F = 4.215, *p* = 0.134).

**TABLE 3:** Daily survival probability and the average life expectancy of the radio-sterilized *Aedes albopictus* males in the field.

Marked sterile males	Trapping method	Probability of daily survival	Average life expectancy (d)
Green	BGS	0.78	4.05
	HLC	0.80	4.55
Orange	BGS	0.76	3.64
	HLC	0.72	3.03
Pink	BGS	0.81	4.69
	HLC	0.84	5.76
Yellow	BGS	0.79	4.31
	HLC	0.78	4.06

BGS, BG-Sentinel trap; HLC, Human Landing Catch.

### Ratio of Sterile to Wild Males

**FIGURE 4:**
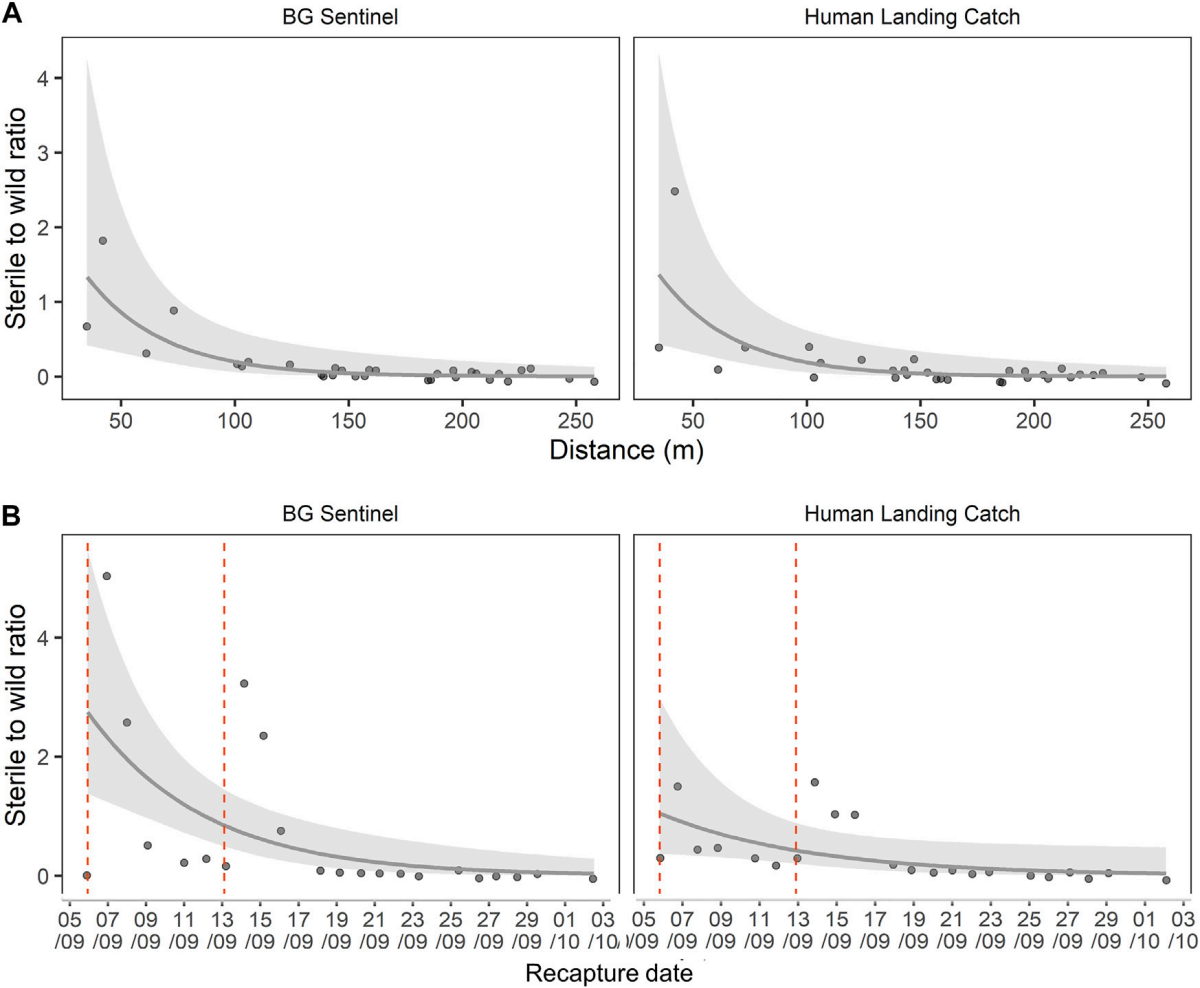
Dynamic of the sterile-to-wild male ratio over the distance from the release point **(A)** and time elapsed since release **(B)**. Vertical dotted lines represent the release dates.

The estimation of daily sterile to wild male ratios during the MRR is shown in Figure 4. The ratio varied with distance from the release point (GLM, df = 1, F = 32.3, *p* < 0.05), but no difference was observed between trapping methods (BGS and HLC) (GLM, df = 1, F = 0.0, *p* = 0.989). The sterile-to-wild ratio varied over time (GLM, df = 1, F = 12.78, *p* < 0.05), while the trapping method had no impact (GLM, df = 1, F = 1.921, *p* = 0.173). The variation in both distance and time showed a similar trend for both BGS and HLC collection methods. The maximum sterile-to-wild male ratio obtained in the release area over time collection was 4.97 sterile for one wild male. The overall mean ratio for the entire study period was 0.45 sterile for one wild male. There was a rapid decrease in the sterile to wild male ratio (within the first week) after release and within 100 m from the release point (Figure 4).

### Wild Male Population Size Estimation

The wild male population size in the area was estimated for the whole collection period using the Lincoln index modified version calculation that corrects for small samples and compensates for daily survival, and the results are presented in Supplementary Table S2. Considering only recapture data from BGS, the Lincoln index estimated the mean population size to be 72,181 males in the overall estimated area of 20 hectares equivalent to 3,609 males/ha. Meanwhile, data from HLC estimated the mean number of males to be 118,691 males (5,934 males/ha).

### Field Competitiveness

**FIGURE 5:**
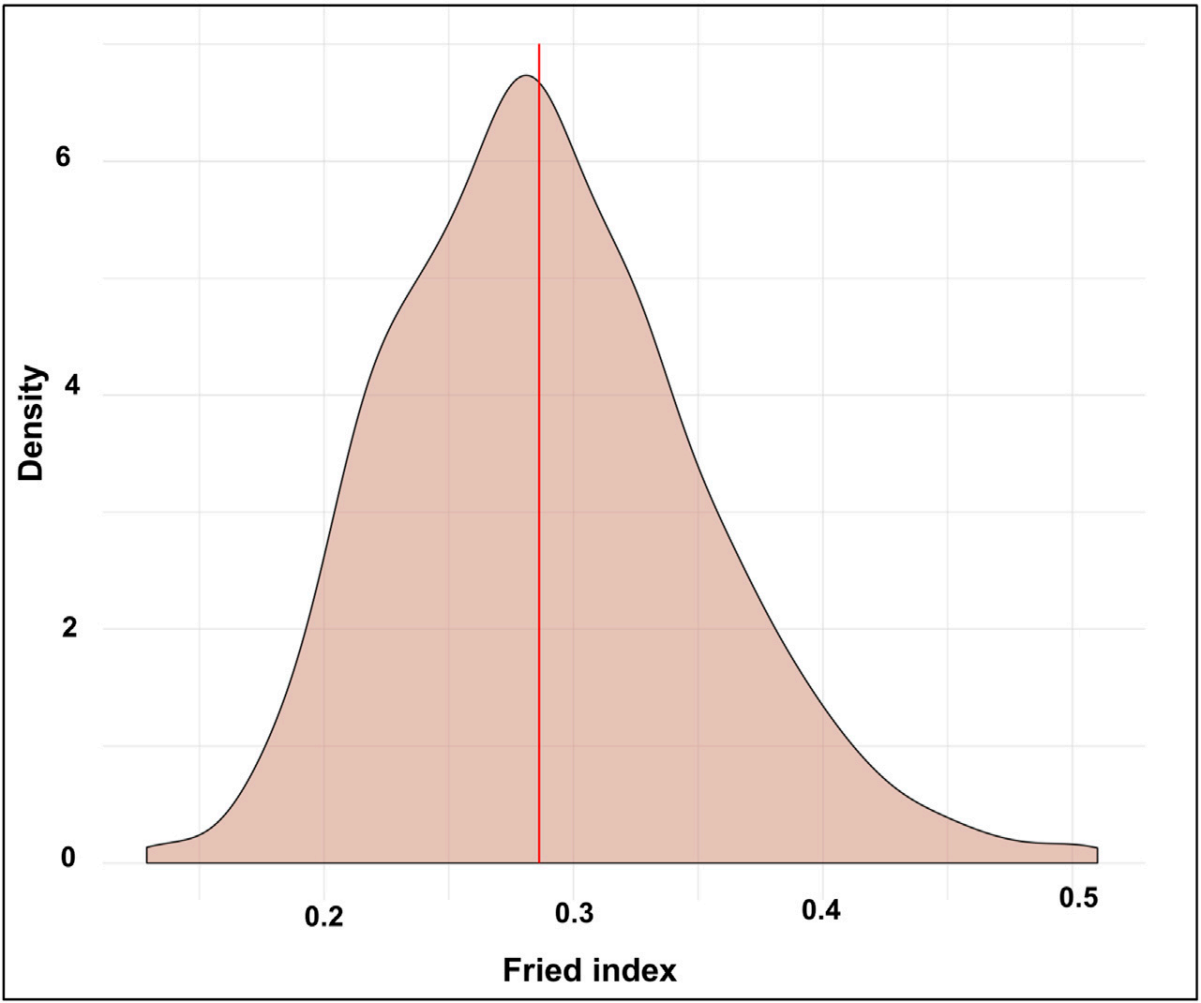
Estimation of the Fried index from 1,000 bootstraps in the distributions of sterile to wild male ratios in traps. The density corresponds to the percentage of the simulations for a given value.

In the three control sites, the natural egg fertility from June to November 2017 ranged from 90% to 100%, and no statistical difference was found among sites (*p* > 0.5). The overall natural fertility calculated in the untreated areas during the MRR study period was 98.24% ± 01.86% (mean ± SD), while in the release site the observed fertility was 74.10 ± 30.85% (mean ± SD). The Fried index estimated from 1,000 bootstraps is presented in Figure 5. The overall Fried Index was evaluated at 0.28% and the 95% confidence interval was [0.19–0.42].

## Discussion

MRR studies are commonly carried out in ecological research in a diverse range of species (McKenzie, 1974; Smith et al., 1999) to assess population size, seasonal dynamics, and dispersal (Harrington et al., 2008; Marini et al., 2010). In our study, MRR allowed us to quantify the dispersal, survival, and competitiveness of irradiated male *Ae. albopictus* in preparation for a field trial in Albania, as recommended for any method based on male release (Bouyer et al., 2020b). Two (series) sessions of MRR were performed with similar recapture rates for both BGS and HLC methods (3.93%) over 2 weeks in which the majority of males were caught within 5 days post-release. This result is consistent with the literature (Bellini et al., 2010) or showed higher recapture rates than in the study by Iyaloo et al. (2020) who found recapture rates in *Ae. albopictus* ranging from 0.8% to 1.3% over 6 days post release and the study by Caputo et al. (2021) that found a recapture rate of 1.8% in the first 6 days. Winskill et al. (2015) found a recapture rate of 0.36% for another genetic control method based on the release of insects carrying a dominant lethal gene (RIDL). Extreme meteorological conditions (temperature, relative humidity, and wind speed) can influence mosquito abundance, survival, and dispersal (Marcantonio et al., 2019). Although environmental conditions were globally suitable during this experiment, rains occurring during the second release might have caused the observed variations between the two series.

Understanding the ability of released sterile male mosquitoes to disperse in an area being targeted by a suppression strategy is crucial for predicting the required release pattern (Winskill et al., 2015). Dispersal defines the capacity of individuals to spread from a fixed or constant source (Gavriel et al., 2012). Generally, male mosquitoes may disperse to find sugar and swarms for mating. There are very few studies that examine the dispersal capacity of male mosquitoes while considering all aspects of the SIT process from production to release. Our study showed an acceptable dispersal capacity when compared to those studies (Lacroix et al., 2009; Le-Goff et al., 2019). Most of the released males were recaptured within 100 m from the release point, but the MDT was 70.78 ± 7.050 m, which is higher than the 52.8 m of RIDL male *Ae. aegypti* (Winskill et al., 2015) and close to the 83 m of irradiated male *Ae. aegypti* mosquitoes released from the ground (Bouyer et al., 2020a), both in Brazil. *Aedes* are typically short-dispersing species (Christophers, 1960), and *Ae. albopictus* males and females’ active dispersal is limited to a few hundred meters (Vavassori et al., 2019). We observed a rapid decrease in the sterile to wild male ratio within the first week after release and within 100 m from the release point, suggesting the need for releasing sterile males more than once a week at a maximum distance of 100 m between release points.

Some of the released males were able to survive up to 15 days after release with a good mean survival (PDS of 0.79 and an ALE of 4.26 days), higher than the results found by Neira et al. (2014) in Panama with non-irradiated marked male *Ae. aegypti* (0.65 and 2.3 days, respectively). Trewin et al. (2021) also found a lower survival for wild male *Ae. aegypti* marked with Rhodamine B (0.55 and 1.69 days, respectively). Their MDT was, however, 295.2 m, much higher than in our study. In Brazil, irradiated male *Ae. aegypti* mosquitoes released from the ground had PDS of 0.20–0.63 (Bouyer et al., 2020a), again lower than in our study.

It is important to note that the sterile males used in our trial were imported via long-distance transportation, following long-distance transportations attempts in Europe (e.g., from CAA laboratory, Bologna, Italy to Germany, Montenegro, and Greece) (Balatsos et al., 2021). Despite 20% of the mortality observed during transport, the released males showed an overall good field quality. Our results show the feasibility of regional production facilities for sterile *Aedes* invasive species in support of newly invaded areas, thus reducing initial risks and costs related to the establishment of mass-rearing production units.

Knowledge of the size of the wild male population of the target area is crucial in determining the dose of sterile males required to achieve the appropriate sterile-to-wild male ratio for controlling the population effectively (Proverbs et al., 1982; Rendón et al., 2004). Here, the male population was as high as 4,700 per hectare, supporting the need of preliminary reduction of the densities with complementary methods such as breeding source reduction, door-to-door (sanitary education), and insecticide-based treatments before starting the SIT component.

We found competitiveness of 0.28 in this study, which is higher than 0.13, the observed average of several pilot trials conducted in Italian urban areas (Bellini et al., 2021). In another MRR study carried out in Brazil, the competitiveness of sterile males released using a release system mounted on an unmanned aerial vehicle (drone) was very close to our results, that is, 0.26 (95% confidence interval, 0.05–0.72) (Bouyer et al., 2020a). However, a higher competitiveness index of 0.5–0.7 with irradiated triple Wolbachia-infected male *Ae. albopictus* was observed in China (Zheng et al., 2019). Our results suggest that the quality of the released mosquitoes was still enough to run a pilot SIT trial in Albania since a competitiveness index value of 0.2 is considered the minimum acceptable threshold (Bouyer et al., 2020a).

Irradiated males are generally considered less competitive than their wild counterparts (Rendón et al., 2004; Bakri et al., 2005; Bellini et al., 2021). An overflooding ratio of 10 is an empirical target in SIT programs (Oliva et al., 2021) but may vary between species: 7:1 for some tsetse fly species (Vreysen, 2005); 60:1 for pink bollworm, *Pectinophora gossypiella* (Walters et al., 1998); 40:1 for codling moth, *Cydia pomonella* (Proverbs et al., 1982); and 100:1 for painted apple moth, *Orgyia anartoides* (Wee et al., 2005; Suckling et al., 2007). Here, we observed an average ratio of 0.45 sterile for one wild male, dropping quickly after the release day. Considering our results, this would require to approximately release 700,000 sterile males weekly in our target area of 20-ha, probably in two releases of half this number and in several release points, to cause a 74% induced sterility. This underlines again the need for a preliminary reduction of the target population using conventional methods such as breeding source reduction or to start the releases at a more appropriate period of the year when the density of the wild population is lower (Douchet et al., 2021).

Our results demonstrated that BGS + CO_2_ and HLC allowed similar estimations of several parameters measured. They also showed comparable efficiency in terms of the number of mosquitoes captured (though during different collection times). To our experience, the variability of catches might greatly increase if trap stations are not chosen by experienced persons. One of the drawbacks of BGS is its price which makes it expensive to purchase and deploy since it is set for 24 h periods covering a full daily mosquito activity period. They also require power, which implies the use of a household electrical outlets or batteries. Their main advantage is to provide a more standardized estimation of mosquito densities. Conversely, HLC is low-cost, performed only within 15 min and if possible, at the high activity periods of *Aedes*, to reduce the noise for statistical analyses. However, HLC data may suffer from variability due to differences in personal attractiveness to mosquitoes as well as individual abilities to catch mosquitoes, making this method more difficult to standardize.

This MRR study was conducted in September only (the end of mosquito season), and the total trapped area was only 250 m of ray which constitutes the main limitations of the study. However, based on the cost-effectiveness of the SIT program and the available data on such MRR studies (Caputo et al., 2021; Lacroix et al., 2009; Balestrino et al., 2022), we believe that the design limit of 250 m seems reasonable to measure the main parameters needed for testing the SIT. Although it provides important insights on the overall performance of the released sterile males, their dispersal and survival in the field as well as the size of the wild population can vary over time during the mosquito season due to variation in environmental conditions. Hence, the release strategies should take into account both the quality and the size of the wild population at different time points of the mosquito season. For reliable estimation of the wild population size over time, an MRR study should be carried out at different time points of the mosquito season (e.g., the beginning, the peak, and/or end of the mosquito season). Furthermore, another limitation of such an MRR study is the lack of non-marked controls in the experimental design. Although of importance, it was, however, not feasible in the present mark-release-recapture study since only the local strain can be released in the study area and there is no biological marker to differentiate non-marked released mosquitoes from the wild ones in order to compare their dispersal and longevity. A recent study conducted in northern Italy (Balestrino et al., 2022) on *Ae. albopictus* under the same rearing and climate conditions but released in the summer (July–August) without long-distance transportation has shown a lower recapture rate (0.27%–1.7%) and PDS 65.4% ± 11.5% to 80.0% ± 6.4%). However, they found much better flight range (FR_50_ = 76.0 ± 35.7 m; FR_90_ = 227.0 ± 74.8 m; MAX = 249.8 ± 45.2 m) and mean distance travelled (126.4 ± 44.6 to 172.9 ± 45.8 m) in comparison to our study. This difference might be due to the transportation and also to the seasonal environmental conditions, highlighting the need to carry out MRR studies at different time points. Nonetheless, a baseline data collection required before every SIT pilot trial following the phased conditional approach, coupled with this MRR study will allow a better estimation of the population abundance and size over time in Albania.”

## Conclusions

Overall, the results of this MRR study allowed us to estimate the minimum distance required between sterile male release points (100 m), as well as the necessary frequency (3–4 days) of releases to be used in future SIT trials in Albania. The release time in the day did not appear as a constraint. Irradiated males dispersed, survived long enough and competed well enough with their wild male counterparts to warrant the feasibility of the next phase, that is, a suppression trial. The main limitations of this study are that it was conducted only in September and should be repeated at the beginning of the mosquito season (April–June) and that the total trapped area was only 250 m of ray. However, our data still provide critical baseline information for better decision-making, designing and elaborating of appropriate planning of SIT pilot suppression studies in Albania and other countries in Europe with similar environmental conditions.

## Data Availability

The original contributions presented in the study are included in the article/Supplementary Material; further inquiries can be directed to the corresponding authors.

## References

[B1] Adhami J. Reiter P. (1998). Introduction and Establishment of *Aedes (Stegomyia) Albopictus* Skuse (Diptera: Culicidae) in Albania. J. Am. Mosq. Control. Assoc. 14, 340–343. 9813831

[B2] Bakran-Lebl K. Zittra C. Harl J. Shahi-Barogh B. Grätzl A. Ebmer D. (2021). Arrival of the Asian Tiger Mosquito, *Aedes albopictus* (Skuse, 1895) in Vienna, Austria and Initial Monitoring Activities. Transbound. Emerg. Dis. 68, 3145–3150. 10.1111/tbed.14169 34051130

[B3] Bakri A. Mehta K. Lance D. (2005). “Sterilizing Insects with Ionizing Radiation,” in Sterile Insect Technique. Editors Dyck V. A. Hendrichs J. Robinson A. S. (Dordrecht, Netherlands: Springer), 233–268. ISBN 1-4020-4050-4.

[B4] Balatsos G. Puggioli A. Karras V. Lytra I. Mastronikolos G. Carrieri M. (2021). Reduction in Egg Fertility of *Aedes albopictus* Mosquitoes in Greece Following Releases of Imported Sterile Males. Insects 12, 110. 10.3390/insects12020110 33513716 PMC7911890

[B5] Balestrino F. Medici A. Candini G. Carrieri M. Maccagnani B. Calvitti M. (2010). γ Ray Dosimetry and Mating Capacity Studies in the Laboratory on *Aedes albopictus* Males. J. Med. Entomol. 47, 581–591. 10.1093/jmedent/47.4.581 20695273 PMC7027263

[B6] Balestrino F. Puggioli A. Bellini R. Petric D. Gilles J. R. L. (2014a). Mass Production Cage forAedes albopictus(Diptera: Culicidae). J. Med. Entomol. 51, 155–163. 10.1603/me13130 24605465

[B7] Balestrino F. Puggioli A. Carrieri M. Bouyer J. Bellini R. (2017). Quality Control Methods for *Aedes albopictus* Sterile Male Production. PLoS Negl. Trop. Dis. 11, e0005881. 10.1371/journal.pntd.0005881 28892483 PMC5608434

[B8] Balestrino F. Puggioli A. Gilles J. R. L. Bellini R. (2014b). Validation of a New Larval Rearing Unit for *Aedes albopictus* (Diptera: Culicidae) Mass Rearing. PLos One 9, e91914. 10.1371/journal.pone.0091914 24647347 PMC3960149

[B9] Balestrino F. Puggioli A. Malfacini M. Albieri A. Carrieri M. Bouyer J. (2022). Field Performance Assessment of *Aedes albopictus* Irradiated Males through Mark-Release-Recapture 2 Trials with Multiple Release Points. Front. Bioeng 10, 876677. 10.3389/fbioe.2022.876677 PMC9344911 35928955

[B10] Becker N. Schön S. Klein A.-M. Ferstl I. Kizgin A. Tannich E. (2017). First Mass Development of *Aedes albopictus* (Diptera: Culicidae)-its Surveillance and Control in Germany. Parasitol. Res. 116, 847–858. 10.1007/s00436-016-5356-z 28116530 PMC5313584

[B11] Bellini R. Albieri A. Balestrino F. Carrieri M. Porretta D. Urbanelli S. (2010). Dispersal and Survival of *Aedes albopictus* (Diptera: Culicidae) Males in Italian Urban Areas and Significance for Sterile Insect Technique Application. Jnl. Med. Entom. 47, 1082–1091. 10.1603/me09154 21175057

[B12] Bellini R. Calvitti M. Medici A. Carrieri M. Celli G. Maini S. (2007). “Use of the Sterile Insect Technique against *Aedes albopictus* in Italy: First Results of a Pilot Trial,” in Area-Wide Control of Insect Pests. Editors Vreysen M. J. B. Robinson A. S. Hendrichs J. (Dordrecht, Netherlands: Springer), 505–515. 10.1007/1978-1001-4020-6059-1005_1047

[B13] Bellini R. Carrieri M. Balestrino F. Puggioli A. Malfacini M. Bouyer J. (2021). Field Competitiveness of *Aedes albopictus* (Diptera: Culicidae) Irradiated Males in Pilot Sterile Insect Technique Trials in Northern Italy. J. Med. Entomol. 58, 807–813. 10.1093/jme/tjaa235 33179753

[B14] Bellini R. Michaelakis A. Petrić D. Schaffner F. Alten B. Angelini P. (2020). Practical Management Plan for Invasive Mosquito Species in Europe: I. Asian Tiger Mosquito *(Aedes albopictus*). Travel Med. Infect. Dis. 35, 101691. 10.1016/j.tmaid.2020.101691 32334085

[B15] Benedict M. Q. Levine R. S. Hawley W. A. Lounibos L. P. (2007). Spread of the Tiger: Global Risk of Invasion by the MosquitoAedes Albopictus. Vector-Borne Zoonotic Dis. 7, 76–85. 10.1089/vbz.2006.0562 17417960 PMC2212601

[B16] Benedict M. Q. Quinlan M. M. Harrington L. Lounibos L. Reisen W. Tabachnick W. (2018). Genetically Engineered Mosquitoes for Pathogen Control. Vector-Borne Zoonotic Dis. 18, 1. 10.1089/vbz.2018.29001.ben 29337663 PMC5846568

[B17] Bimbilé Somda N. S. Maïga H. Mamai W. Yamada H. Ali A. Konczal A. (2019). Insects to Feed Insects - Feeding Aedes Mosquitoes with Flies for Laboratory Rearing. Sci. Rep. 9, 11403. 10.1038/s41598-019-47817-x 31388041 PMC6684809

[B18] Bouyer J. Culbert N. J. Dicko A. H. Pacheco M. G. Virginio J. Pedrosa M. C. (2020a). Field Performance of Sterile Male Mosquitoes Released from an Uncrewed Aerial Vehicle. Sci. Robot. 5, eaba6251. 10.1126/scirobotics.aba6251 33022616

[B21] Bouyer J. Yamada H. Pereira R. Bourtzis K. Vreysen M. J. B. (2020b). Phased Conditional Approach for Mosquito Management Using Sterile Insect Technique. Trends Parasitol. 36, 325–336. 10.1016/j.pt.2020.01.004 32035818

[B22] Buonaccorsi J. P. Harrington L. C. Edman J. D. (2003). Estimation and Comparison of Mosquito Survival Rates with Release-Recapture-Removal Data. J. Med. Entomol. 40, 6–17. 10.1603/0022-2585-40.1.6 12597647

[B93] Caputo P. Langella G. Petrella V. Virgillito C. Manica M. Filipponi F. (2021). Aedes Albopictus Bionomics Data Collection by Citizen Participation on Procida Island, a Promising Mediterranean Site for the Assessment of Innovative and Community-Based Integrated Pest Management Methods. PLoS. Negl. Trop. Dis. 15, 1–29. 10.1371/journal.pntd.0009698 PMC8445450 34529653

[B23] Christophers S. (1960). The Yellow Fever Mosquito-Aedes aegypti (Linnaeus). London: Cambridge University Press.

[B24] Culbert N. J. Balestrino F. Dor A. Herranz G. S. Yamada H. Wallner T. (2019). Author Correction: A Rapid Quality Control Test to Foster the Development of Genetic Control in Mosquitoes. Sci. Rep. 9, 8427. 10.1038/s41598-019-44071-z 31165740 PMC6549155

[B25] Culbert N. J. Kaiser M. Venter N. Vreysen M. J. B. Gilles J. R. L. Bouyer J. (2020). A Standardised Method of Marking Male Mosquitoes with Fluorescent Dust. Parasites Vectors 1513 (1), 192. 10.1186/s13071-020-04066-6 PMC7158013 32293537

[B26] Cvetkovikj A. Djadjovski I. Krstevski K. Popova Z. Rashikj L. Atanasova K. (2020). New Records of the Asian Tiger Mosquito (*Aedes albopictus*) in North Macedonia. Mac. Vet. Rev. 43 (2), 125–129. 10.2478/macvetrev-2020-0025

[B27] Douchet L. Haramboure M. Baldet T. L'Ambert G. Damiens D. Gouagna L. C. (2021). Comparing Sterile Male Releases and Other Methods for Integrated Control of the Tiger Mosquito in Temperate and Tropical Climates. Sci. Rep. 11, 7354–7414. 10.1038/s41598-021-86798-8 33795801 PMC8016901

[B28] Dyck V. A. Reyes Flores J. Vreysen M. J. B. Regidor Fernández E. E. Barnes B. N. Loosjes M. (2021). “Management of Area-Wide Pest Management Programmes that Integrate the Sterile Insect Technique,” in Sterile Insect Technique. Principles and Practice in Area-Wide Integrated Pest Management© 2021 IAEA. Second Edition (Boca Raton, Florida, USA: CRC Press), 781–814. 10.1201/9781003035572-24

[B29] ECDC (2019). Epidemiological Update: Third Case of Locally Acquired Zika Virus Disease in Hyères, France. Available at: https://www.ecdc.europa.eu/en/news-events/epidemiological-update-third-case-locally-acquired-zika-virus-disease-hyeres-france .

[B30] Efron B. (1979). Bootstrap Methods: Another Look at the Jacknife. Ann. Stat. 7, 1–26. 10.1214/aos/1176344552

[B31] Epopa P. S. Millogo A. A. Collins C. M. North A. Tripet F. Benedict M. Q. (2017). The Use of Sequential Mark-Release-Recapture Experiments to Estimate Population Size, Survival and Dispersal of Male Mosquitoes of the *Anopheles gambiae* Complex in Bana, a West African Humid Savannah Village. Parasites Vectors 10, 376. 10.1186/s13071-017-2310-6 28784147 PMC5547516

[B19] FAO/IAEA (2020a). Editors J. Bouyer F. Balestrino N. Culbert Y. Yamada R. Argilés (Vienna, Austria: Food and Agriculture Organization of the United Nations/International Atomic Energy Agency), 22. Version 1.0.Guidelines for Mark-Release-Recapture Procedures of Aedes Mosquitoes.

[B32] FAO/IAEA (2020b). Guidelines for Mass-Rearing of *Aedes* Mosquitoes. Vienna, Austria: Food and Agriculture Organization of the United Nations/International Atomic Energy Agency, 24. Version 1.0.

[B33] Farajollahi A. Kesavaraju B. Price D. C. Williams G. M. Healy S. P. Gaugler R. (2009). Field Efficacy of BG-Sentinel and Industry-Standard Traps for *Aedes albopictus* (Diptera: Culicidae) and West Nile Virus Surveillance. me 46, 919–925. 10.1603/033.046.0426 19645298

[B34] Fried M. (1971). Determination of Sterile-Insect Competitiveness. J. Econ. Entomol. 64, 869–872. 10.1093/jee/64.4.869

[B35] Gavriel S. Gazit Y. Leach A. Mumford J. Yuval B. (2012). Spatial Patterns of Sterile Mediterranean Fruit Fly Dispersal. Entomol. Exp. Appl. 142, 17–26. 10.1111/j.1570-7458.2011.01197.x

[B36] Gillies M. T. (1961). Studies on the Dispersion and Survival of *Anopheles gambiae* Giles in East Africa, by Means of Marking and Release Experiments. Bull. Entomol. Res. 52, 99–127. 10.1017/s0007485300055309

[B37] Grigoraki L. Puggioli A. Mavridis K. Douris V. Montanari M. Bellini R. (2017). Striking Diflubenzuron Resistance in *Culex pipiens*, the Prime Vector of West Nile Virus. Sci. Rep. 7, 11699. 10.1038/s41598-017-12103-1 28916816 PMC5601912

[B38] Hagler J. R. Jackson C. G. (2001). Methods for Marking Insects: Current Techniques and Future Prospects. Annu. Rev. Entomol. 46, 511–543. 10.1146/annurev.ento.46.1.511 11112178

[B39] Harrington L. C. Buonaccorsi J. P. Edman J. D. Costero A. Kittayapong P. Clark G. G. (2001). Analysis of Survival of Young and OldAedes aegypti(Diptera: Culicidae) from Puerto Rico and Thailand. J. Med. Entomol. 38, 537–547. 10.1603/0022-2585-38.4.537 11476334

[B40] Harrington L. C. Ponlawat A. Edman J. D. Scott T. W. Vermeylen F. (2008). Influence of Container Size, Location, and Time of Day on Oviposition Patterns of the Dengue Vector,*Aedes aegypti*, in Thailand. Vector-Borne Zoonotic Dis. 8, 415–424. 10.1089/vbz.2007.0203 18279006 PMC2978047

[B41] Hemingway J. Ranson H. (2000). Insecticide Resistance in Insect Vectors of Human Disease. Annu. Rev. Entomol. 45, 371–391. 10.1146/annurev.ento.45.1.371 10761582

[B42] Iyaloo D. P. Facknath S. Bheecarry A. (2020). A Mark-Release-Recapture Experiment with Radio-Sterilised *Aedes albopictus* Males as Part of a Sterile Insect Technique Programme against the Vector Mosquito in Panchvati, Mauritius. Afr. Entomol. 28, 187–191. 10.4001/003.028.0187

[B43] Kampen H. Kronefeld M. Werner D. (2012). “Culicid Mosquitoes as Vectors of Disease Agents in Europe,” in Arthropods as Vectors of Emerging Diseases. Editor Mehlhorn H. (Düsseldorf, Germany: Springer), 1–30. 10.1007/978-3-642-28842-5_1

[B44] Kolimenakis A. Latinopoulos D. Bithas K. Richardson C. Lagouvardos K. Stefopoulou A. (2019). Exploring Public Preferences, Priorities, and Policy Perspectives for Controlling Invasive Mosquito Species in Greece. TropicalMed 4, 83. 10.3390/tropicalmed4020083 PMC6632057 31109075

[B45] Lacroix R. Delatte H. Hue T. Reiter P. (2009). Dispersal and Survival of Male and FemaleAedes albopictus(Diptera: Culicidae) on Réunion Island. J. Med. Entomol. 46, 1117–1124. 10.1603/033.046.0519 19769043

[B46] Le Goff G. Damiens D. Ruttee A.-H. Payet L. Lebon C. Dehecq J.-S. (2019). Field Evaluation of Seasonal Trends in Relative Population Sizes and Dispersal Pattern of *Aedes albopictus* Males in Support of the Design of a Sterile Male Release Strategy. Parasites Vectors 12, 81. 10.1186/s13071-019-3329-7 30755268 PMC6371565

[B47] Lounibos L. P. (2002). Invasions by Insect Vectors of Human Disease. Annu. Rev. Entomol. 47, 233–266. 10.1146/annurev.ento.47.091201.145206 11729075

[B48] Maïga H. Bimbilé-Somda N. S. Yamada H. Wood O. Damiens D. Mamai W. (2017). Enhancements to the Mass-Rearing Cage for the Malaria Vector, *Anopheles Arabiensis* for Improved Adult Longevity and Egg Production. Entomol. Exp. Appl. 164, 269–275. 10.1111/eea.12614

[B49] Maïga H. Damiens D. Diabaté A. Dabiré R. K. Ouédraogo G. A. Lees R. S. (2016). Large-scale *Anopheles Arabiensis* Egg Quantification Methods for Mass-Rearing Operations. Malar. J. 15, 72. 10.1186/s12936-016-1119-7 26852018 PMC4744385

[B50] Maïga H. Mamai W. Bimbilé Somda N. S. Konczal A. Wallner T. Herranz G. S. (2019). Reducing the Cost and Assessing the Performance of a Novel Adult Mass-Rearing Cage for the Dengue, Chikungunya, Yellow Fever and Zika Vector, *Aedes aegypti* (Linnaeus). PLOS Negl. Trop. Dis. 13, e0007775. 10.1371/journal.pntd.0007775 31553724 PMC6779276

[B51] Mamai W. Bimbilé Somda N. S. Maiga H. Konczal A. Wallner T. Bakhoum M. T. (2019b). Black Soldier Fly (*Hermetia Illucens*) Larvae Powder as a Larval Diet Ingredient for Mass-Rearing *Aedes* Mosquitoes. Parasite 26, 57. 10.1051/parasite/2019059 31535969 PMC6752115

[B52] Mamai W. Bimbile-Somda N. S. Maiga H. Juarez J. G. Muosa Z. A. I. Ali A. B. (2017). Optimization of Mosquito Egg Production under Mass Rearing Setting: Effects of Cage Volume, Blood Meal Source and Adult Population Density for the Malaria Vector, *Anopheles Arabiensis* . Malar. J. 16, 41. 10.1186/s12936-017-1685-3 28118825 PMC5260048

[B53] Mamai W. Maiga H. Gárdos M. Bán P. Bimbilé Somda N. S. Konczal A. (2019a). The Efficiency of a New Automated Mosquito Larval Counter and its Impact on Larval Survival. Sci. Rep. 9, 7413. 10.1038/s41598-019-43333-0 31092868 PMC6520403

[B54] Marcantonio M. Reyes T. Barker C. (2019). Quantifying *Aedes aegypti* Dispersal in Space and Time: a Modeling Approach. Ecosphere 10, e02977. 10.1002/ecs2.2977

[B55] Marini F. Caputo B. Pombi M. Tarsitani G. Della Torre A. (2010). Study ofAedes Albopictusdispersal in Rome, Italy, Using Sticky Traps in Mark-Release-Recapture Experiments. Med. Vet. Entomol. 24, 361–368. 10.1111/j.1365-2915.2010.00898.x 20666995

[B56] McKenzie J. A. (1974). The Distribution of Vineyard Populations of *Drosophila melanogaster* and *Drosophila simulans* during Vintage and Non-vintage Periods. Oecologia 15, 1–16. 10.1007/bf00345225 28308614

[B57] Medici A. Carrieri M. Scholte E.-J. Maccagnani B. Dindo M. L. Bellini R. (2011). Studies on *Aedes albopictus* Larval Mass-Rearing Optimization. Jnl. Econ. Entom. 104, 266–273. 10.1603/ec10108 21404867

[B58] Medlock J. M. Hansford K. M. Schaffner F. Versteirt V. Hendrickx G. Zeller H. (2012). A Review of the Invasive Mosquitoes in Europe: Ecology, Public Health Risks, and Control Options. Vector-Borne Zoonotic Dis. 12, 435–447. 10.1089/vbz.2011.0814 22448724 PMC3366101

[B59] Medlock J. M. Hansford K. M. Versteirt V. Cull B. Kampen H. Fontenille D. (2015). An Entomological Review of Invasive Mosquitoes in Europe. Bull. Entomol. Res. 105, 637–663. 10.1017/s0007485315000103 25804287

[B60] Muir L. E. Kay B. H. (1998). *Aedes aegypti* Survival and Dispersal Estimated by Mark-Release-Recapture in Northern Australia. Am. J. Trop. Med. Hyg. 58 (3), 277–282. 10.4269/ajtmh.1998.58.277 9546403

[B61] Neira M. Lacroix R. Cáceres L. Kaiser P. E. Young J. Pineda L. (2014). Estimation of *Aedes aegypti* (Diptera: Culicidae) Population Size and Adult Male Survival in an Urban Area in Panama. Mem. Inst. Oswaldo Cruz 109, 879–886. 10.1590/0074-0276140136 25410991 PMC4296492

[B62] Oliva C. F. Benedict M. Q. Collins C. M. Baldet T. Bellini R. Bossin H. (2021). Sterile Insect Technique (SIT) against *Aedes* Species Mosquitoes: A Roadmap and Good Practice Framework for Designing, Implementing and Evaluating Pilot Field Trials. Insects 12, 191. 10.3390/insects12030191 33668374 PMC7996155

[B64] Pichler V. Bellini R. Veronesi R. Arnoldi D. Rizzoli A. Lia R. P. (2018). First Evidence of Resistance to Pyrethroid Insecticides in Italian *Aedes albopictus* Populations 26 Years after Invasion. Pest. Manag. Sci. 74, 1319–1327. 10.1002/ps.4840 29278457

[B65] Pollock K. H. (1991). Review Papers: Modeling Capture, Recapture, and Removal Statistics for Estimation of Demographic Parameters for Fish and Wildlife Populations: Past, Present, and Future. J. Am. Stat. Assoc. 86, 225–238. 10.1080/01621459.1991.10475022

[B66] Proverbs M. D. Newton J. R. Campbell C. J. (1982). Codling Moth: A Pilot Program of Control by Sterile Insect Release in British Columbia. Can. Entomol. 114, 363–376. 10.4039/ent114363-4

[B67] Rendón P. McInnis D. Lance D. Stewart J. (2004). Medfly (Diptera:Tephritidae) Genetic Sexing: Large-Scale Field Comparison of Males-Only and Bisexual Sterile Fly Releases in Guatemala. ec 97, 1547–1553. 10.1603/0022-0493-97.5.1547 15568342

[B68] Romeis J. Collatz J. Glandorf D. C. M. Bonsall M. B. (2020). The Value of Existing Regulatory Frameworks for the Environmental Risk Assessment of Agricultural Pest Control Using Gene Drives. Environ. Sci. Policy 108, 19–36. 10.1016/j.envsci.2020.02.016

[B69] Schoener E. Zittra C. Weiss S. Walder G. Barogh B. S. Weiler S. (2019). Monitoring of Alien Mosquitoes of the Genus *Aedes* (Diptera: Culicidae) in Austria. Parasitol. Res. 118, 1633–1638. 10.1007/s00436-019-06287-w 30877440 PMC6478629

[B70] Scholte E. Schaffner F. (2007). “Waiting for the Tiger: Establishment and Spread of the *Aedes albopictus* Mosquito in Europe,” in Emerging Pests and Vector-Borne Diseases in Europebook Series Ecology and Control of Vector-Borne Diseases. Editors Takken W. Knols B. G. J. (Wageningen, Netherlands: Wageningen Academic Publishers), 241–260.

[B71] Silver J. (2007). Mosquito Ecology: Field Sampling Methods. New York: Springer Science and Business Media.

[B72] Smith T. D. Allen J. Clapham P. J. Hammond P. S. Katona S. Larsen F. (1999). An Ocean-basin-wide Mark-Recapture Study of the North Atlantic Humpback Whale (Megaptera Novaeangliae). Mar. Mammal. Sci. 15, 1–32. 10.1111/j.1748-7692.1999.tb00779.x

[B73] Staunton K. M. Crawford J. E. Cornel D. Yeeles P. Desnoyer M. Livni J. (2020). Environmental Influences on *Aedes aegypti* Catches in Biogents Sentinel Traps during a Californian “Rear and Release” Program: Implications for Designing Surveillance Programs. PLoS Negl. Trop. Dis. 14, e0008367. 10.1371/journal.pntd.0008367 32530921 PMC7314095

[B74] Suckling D. Barrington A. Chhagan A. Stephens A. Burnip G. Charles J. (2007). “Eradication of the Australian Painted Apple Moth *Teia Anartoides* in New Zealand: Trapping, Inherited Sterility, and Male Competitiveness,” in Area-Wide Control of Insect Pests: From Research to Field Implementation. Editors Vreysen M. J. B. Robinson A. S. Hendrichs J. (Dordrecht: Springer), 603–615.

[B75] Șuleșco T. Bușmachiu G. Lange U. Schmidt-Chanasit J. Lühken R. (2021). The First Record of the Invasive Mosquito Species *Aedes albopictus* in Chişinӑu, Republic of Moldova, 2020. Parasit. Vectors 14, 565. 10.1186/s13071-021-05060-2 PMC8565072 34732241

[B76] Tisseuil C. Velo E. Bino S. Kadriaj P. Mersini K. Shukullari A. (2018). Forecasting the Spatial and Seasonal Dynamic of *Aedes albopictus* Oviposition Activity in Albania and Balkan Countries. PLoS Negl. Trop. Dis. 12, e0006236. 10.1371/journal.pntd.0006236 29432489 PMC5825170

[B77] Tolle M. A. (2009). Mosquito-borne Diseases. Curr. Problems Pediatr. Adolesc. Health Care 39, 97–140. 10.1016/j.cppeds.2009.01.001 19327647

[B78] Trewin B. J. Pagendam D. E. Johnson B. J. Paton C. Snoad N. Ritchie S. A. (2021). Mark-release-recapture of Male *Aedes aegypti* (Diptera: Culicidae): Use of Rhodamine B to Estimate Movement, Mating and Population Parameters in Preparation for an Incompatible Male Program. PLoS Negl. Trop. Dis. 15, 1–21. 10.1371/journal.pntd.0009357 PMC8183986 34097696

[B79] Vavassori L. Saddler A. Müller P. (2019). Active Dispersal of *Aedes albopictus*: a Mark-Release-Recapture Study Using Self-Marking Units. Parasit. Vectors 12, 583. 10.1186/s13071-019-3837-5 31831040 PMC6909613

[B80] Vermeulen T. Reimerink J. Reusken C. Giron S. de Vries P. (2020). Autochthonous Dengue in Two Dutch Tourists Visiting Département Var, Southern France. Euro Surveill. 39, 2001670. 10.2807/1560-7917.ES.2020.25.39.2001670 PMC7531074 33006305

[B81] Vontas J. Kioulos E. Pavlidi N. Morou E. Della Torre A. Ranson H. (2012). Insecticide Resistance in the Major Dengue Vectors *Aedes albopictus* and *Aedes aegypti* . Pest Biochem. Physiol. 104, 126–131. 10.1016/j.pestbp.2012.05.008

[B82] Vreysen M. (2005). “Monitoring Sterile and Wild Insects in Area-wide Integrated Pest Management Programmes,” in Sterile Insect Technique. Editors Dyck V. A. Hendrichs J. Robinson A. S. (Dordrecht, Netherlands: Springer), 325–361.

[B83] Vreysen M. Saleh K. Ali M. Abdulla A. Zhu Z. Juma K. (2000). *Glossina Austeni* (Diptera: Glossinidae) Eradicated on the Island of Unguja, Zanzibar, Using the Sterile Insect Technique. J. Econ. Entomol. 93, 123–135. 10.1603/0022-0493-93.1.123 14658522

[B84] Walters M. Staten R. Roberson R. (1998). “Pink Bollworm Integrated Management Technology under Field Trial Conditions in the Imperial Valley, California, Pp 1282-1985,” in Proceedings Beltwide Cotton Conferences, Memphis, Tennessee. Editors Dugger P. Richer D. A. (USA: National Cotton Council of America. Nashville, Tennessee).

[B85] Wee S. Suckling D. Burnip G. Hackett J. Barrington A. Pedley R. (2005). Effects of Sub-sterilizing Doses of Gamma Radiation on Adult Longevity and Level of Inherited Sterility in Teia Anartoides (Lepidoptera: Lymantriidae). J. Econ. Entomol. 98, 732–738. 10.1603/0022-0493-98.3.732 16022300

[B86] WHO (2017). Framework for a National Vector Control Needs Assessment. Geneva: World Health Organization, 48.

[B87] WHO/IAEA (2020). Guidance Framework for Testing the Sterile Insect Technique (SIT) as a Vector Control Tool against *Aedes*-Borne Diseases. Geneva & Vienna: World Health Organization, 190.

[B88] Williams C. Long S. Russell R. Ritchie S. (2006). Field Efficacy of the BG-Sentinel Compared with CDC Backpack Aspirators and CO2-baited EVS Traps for Collection of Adult *Aedes aegypti* in Cairns, Queensland, Australia. J. Am. Mosq. Control. Assoc. 22, 296–300. 10.2987/8756-971x(2006)22[296:feotbc]2.0.co;2 17019776

[B89] Winskill P. Carvalho D. O. Capurro M. Alphey L. Donnelly C. McKemey A. (2015). Dispersal of Engineered Male *Aedes aegypti* Mosquitoes. PLoS Negl. Trop. Dis. 9, e0004156. 10.1371/journal.pntd.0004156 26554922 PMC4640874

[B90] Yamada H. Maiga H. Juarez J. Carvalho D. Mamai W. Ali A. (2019). Identification of Critical Factors that Significantly Affect the Dose Response in Mosquitoes Irradiated as Pupae. Parasit. Vectors 12, 435. 10.1186/s13071-019-3698-y 31500662 PMC6734225

[B91] Zheng M. Zhang D. Damiens D. Lees R. Gilles J. (2015). Standard Operating Procedures for Standardized Mass Rearing of the Dengue and Chikungunya Vectors *Aedes aegypti* and *Aedes albopictus* (Diptera: Culicidae) - II - Egg Storage and Hatching. Parasit. Vectors. 8, 348. 10.1186/s13071-015-0951-x 26112698 PMC4485872

[B92] Zheng X. Zhang D. Li Y. Yang C. Wu Y. Liang X. (2019). Incompatible and Sterile Insect Techniques Combined Eliminate Mosquitoes. Nature 572, 56–61. 10.1038/s41586-019-1407-9 31316207

