# Supplementary material for: A Mark-Release-Recapture Study to Estimate Field Performance of Imported Radio-Sterilized Male Aedes albopictus in Albania
Source: Front Bioeng Biotechnol. 2022 Aug 16;10:833698. doi: 10.3389/fbioe.2022.833698 (PMC9424856; doi:10.3389/fbioe.2022.833698)
Supplement: Supplementary file 1 [file DataSheet1.doc]

SUPPLEMENATRY MATERIAL

**SUPPLEMENTARY MATERIAL S1: PROCEDURES FOR THE DETERMINATION OF FERTILITY**

***S1-A- PROTOCOL FOR EGG EMBRYONATION***

Eggs collected in ovitraps are initially placed in plastic containers over sheets of absorbent paper to remove the excess of water and then counted directly on the substrata (germination paper) under a stereomicroscope 20-30X. Stripes of oviposition paper containing *Aedes albopictus* eggs were then cut out, placed inside a 50 ml Falcon tubes and left with the lid unscrewed on it (air can enter and humidity can escape) for one day. Once no water remains in the tubes but paper remains humid, the tubes were sealed and maintained at room condition (23 °C) for seven days before applying the hatching procedure.

***S1-B- PROTOCOL FOR EGG HATCHING***

The egg fertility rate was measured by hatching the egg collected using the following standardized protocol. The different Falcon tubes used to maturate the field collected eggs (see S1-A above) were used as hatching container. A given volume of hatching solution is prepared in the afternoon (18:00) according to the number of Falcon tubes. It consists of a solution of dechlorinated water with 0.33 g/l of Nutrient Broth (CM0001 Oxoid Ltd., Hampshire, England) and 0.07 g/l of Brewer’s yeast (YBD-1KG, Sigma-Aldrich, St. Louis, MO) (Balestrino *et al*. 2010, Zheng *et al*. 2015). The Falcon tubes containing the egg stripes are filled with the solution, tightly sealed and maintained overnight at lab condition (ca. 24 °C). The leftover solution should not be used in the following days.

1) The following day the tubes can be open and the *Ae. albopictus* eggs will be discriminated and assigned to three possible categories: 1-intact eggs; 2-hatched eggs; 3-collapsed eggs.

The eggs normally shaped need to be double checked by squeezing them with a mounted needle under a stereomicroscope to ascertain the presence of the embryo. Embryonated or hatched eggs has to be considered fertile while empty or collapsed eggs can be counted as sterile.

**SUPPLEMENTARY MATERIAL S2: PROTOCOL FOR MARKING ADULT MOSQUITO WITH FLUORESCENT PIGMENTS**

About 2,000 male pupae in a plastic Petri dish are introduced in the cardboard box (length x width x height: 12 x 12 x 18 cm; volume 2.6 litres) for emergence. The top side of the box is covered with white net. One feeder with 10% sucrose solution is placed on the top of each box as energy source for adult males. The dust fluorescent pigment (RADIANT® COLOR N.V. JST44 ORANGE-RED, M5399 dust, Houthalen, Belgium) is applied (maximum 0.30 g/box) to 3 to 4 days old males by manual insufflators (Hygienic vaginal doushe, Farlin® Industrial Co., Taiwan) through the net. In order to accelerate male movement through the created fluorescent powder mist, the box will be gently shaken during next two minutes. Dusted males are released immediately. Males not taking flight in 30 minutes are considered as dead and counted. A sample of about 300 males is randomly withdrawn from several boxes and examined under a stereomicroscope for coloration assigning marked and not marked).

**SUPPLEMENTARY MATERIAL FIGURE S1: Variations in daily mean temperature, relative humidity and daily precipitation during the mark-release-recapture period.**


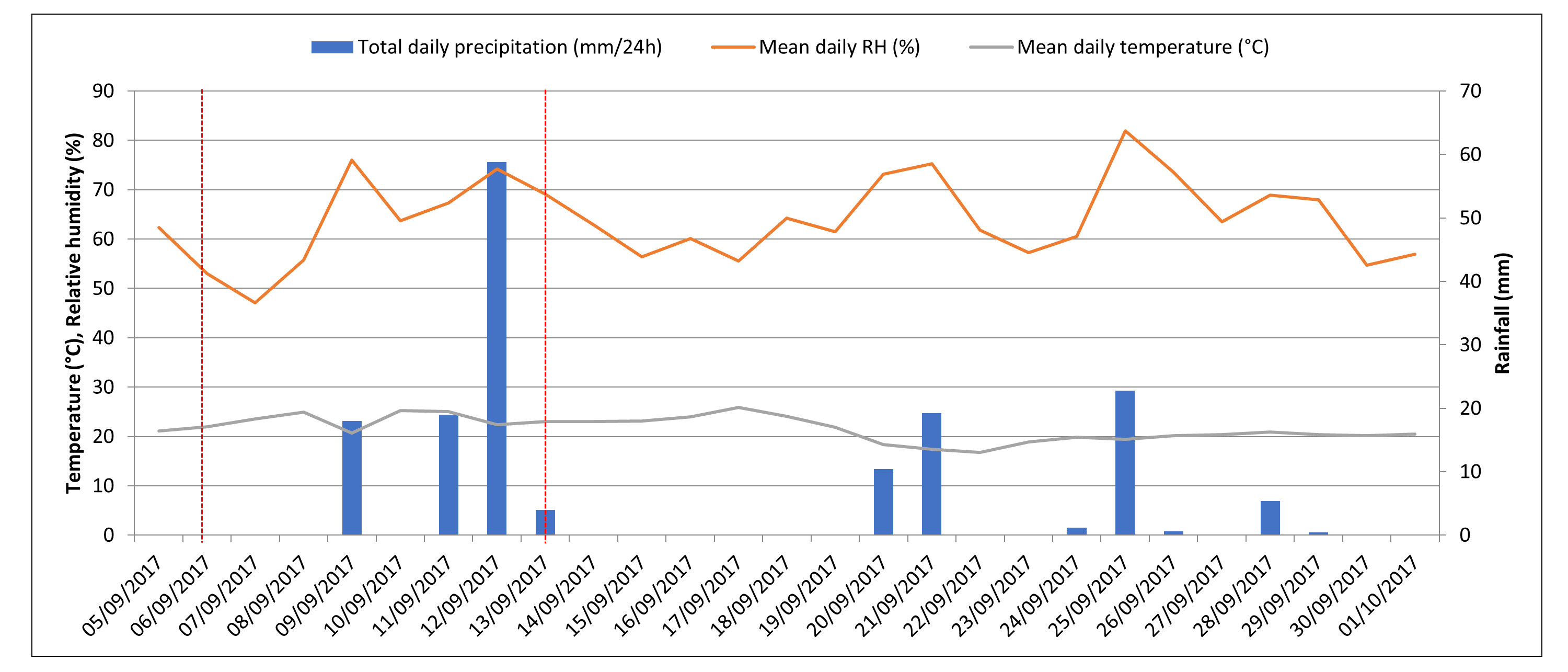


**SUPPLEMENTARY MATERIAL Table S1. Environmental conditions (daily precipitation, daily minimum, maximum and mean temperatures, mean daily temperature and mean daily relative humidity) through the MRR period**.

|  | **Date** | **Total daily precipitation (mm)** | **Mean daily RH  (%)** | **Daily min temp (°C)** | **Daily max temp (°C)** | **Mean daily temp (°C)** | **Mean daily wind speed (m/s)** |
| --- | --- | --- | --- | --- | --- | --- | --- |
|  |  |  |  |  |  |  |  |
|  | 05/09/2017 | 0.0 | 62.3 | 16.1 | 26.0 | 21.1 | 1.3 |
| **MRR 1** | 06/09/2017 | 0.0 | 53.0 | 16.8 | 27.1 | 21.9 | 1.4 |
|  | 12/09/2017 | 58.8 | 74.2 | 17.8 | 27.0 | 22.4 | 1.5 |
| **MRR 2** | 13/09/2017 | 4.0 | 69.1 | 19.1 | 27.0 | 23.1 | 1.4 |

**SUPPLEMENTARY MATERIAL Table S2: Estimation number of the male population in the release area following the modified Lincoln index. BGS = BG-Sentinel trap; HLC = Human Landing Catch.**

| **Trap type** | **Color** | **Lincoln Index modified** |
| --- | --- | --- |
| **BGS** | Green | 91937.98 |
| **BGS** | Orange | 65895.8 |
| **BGS** | Pink | 66494.02 |
| **BGS** | Yellow | 69114.31 |
| **HLC** | Green | 224494.1 |
| **HLC** | Orange | 124774.1 |
| **HLC** | Pink | 93704.68 |
| **HLC** | Yellow | 100576.2 |
